# A missense mutation in *TCN2* is associated with decreased risk for congenital heart defects and may increase cellular uptake of vitamin B12 via Megalin

**DOI:** 10.18632/oncotarget.19377

**Published:** 2017-07-19

**Authors:** Peiqiang Li, Lijuan Huang, Yufang Zheng, Xuedong Pan, Rui Peng, Yueming Jiang, Richard H. Finnell, Haijie Li, Bin Qiao, Hong-Yan Wang

**Affiliations:** ^1^ Obstetrics and Gynecology Hospital, State Key Laboratory of Genetic Engineering at School of Life Sciences, Institute of Reproduction and Development, Fudan University, Shanghai 200011, China; ^2^ Institute of Genetics, School of Basic Medical Sciences, Lanzhou University, Lanzhou City 730000, China; ^3^ Key Laboratory of Reproduction Regulation of NPFPC, Collaborative Innovation Center of Genetics and Development, Fudan University, Shanghai 200032, China; ^4^ Department of Pediatrics, University of Texas at Austin Dell Medical School, Austin, TX 78701, USA; ^5^ Collaborative Innovation Center for Genetics and Development, School of Life Sciences, Fudan University, Jiangwan Campus, Shanghai 200438, China; ^6^ Institute of Cardiovascular Disease, General Hospital of Jinan Military Region, Jinan 250022, China; ^7^ Children's Hospital Fudan University, Shanghai 201102, China

**Keywords:** congenital heart defects, vitamin B12, TCN2, holo-TC, LRP2

## Abstract

Deregulation of folate and vitamin B12 (VB12) metabolism contributes to the risk of congenital heart defects (CHDs). Transcobalamin (TCN2) is essential for transporting VB12 from blood to cells as TCN2-bound VB12 (holo-TC) is the only form for somatic cellular uptake. In this study, we performed an association study between common polymorphisms in 46 one carbon metabolism genes and CHD in 412 CHDs and 213 controls. Only two significant association signals in coding regions were identified: FTCD c.1470C>T & TCN2 c.230A>T. The only missense mutation, TCN2 c.230A>T, was further validated in 412 CHDs and 1177 controls. TCN2 c.230T is significantly associated with reduced CHD risk in North Chinese (odds ratio = 0.67, *P* = 4.62e-05), compared with the 230A allele. Interestingly, the mean level of plasma holo-TC in women with the TA genotype was 1.77-fold higher than that in women with the AA genotype. Further analysis suggested that c.230A>T enhanced the cellular uptake of holo-TC via the LRP2 receptor. Our results determined that a functional polymorphism in TCN2 contributes to the prevalence of CHDs. TCN2 c.230A>T is significantly associated with a reduced CHD risk, likely due to TCN2 c.230T improving the interaction between holo-TC and its LRP2 receptor.

## INTRODUCTION

Congenital heart defects (CHDs) are the most common major human structural birth defects, affecting 8–9 per 1,000 live births worldwide. CHDs are a spectrum of malformations that are anatomically, epidemiologically, developmentally, and clinically heterogeneous [1]. The etiology of CHDs is still not very clear. The genetic variants in CHD patients, the maternal lifestyle, and environmental exposures to teratogens during pregnancy all produce a complex effect on genotype-phenotype correlations with respect to the etiology of CHDs [2, 3].

Studies have shown that maternal nutritional supply such as folic acid has profound impact on fetal development. Based on clinical evidence accumulated over the past few decades, maternal folic acid supplementation could significantly reduce the incidence of congenital malformations, including a 28% reduction in CHDs [4]. However, the mechanism by which folate insufficiency negatively impacts normal cardiac development is still elusive.

As an essential vitamin, folate is critical for many key physiological processes, including one carbon metabolism, DNA/RNA synthesis, and DNA repair and transmethylation reactions. One key metabolite in folate metabolism is tetrahydrofolate (THF), which functions as an important one-carbon donor molecule. Methionine synthase (MTR) catalyzes 5-methyltetrahydrofolate (N^5^-MeTHF) to regenerate THF, while transferring a methyl group to homocysteine (Hcy) to form methionine (Met). This is also the critical step for Hcy biotransformation [5]. Defects in Hcy removal will result in elevated plasma Hcy levels and even induce hyperhomocysteinemia when plasma tHcy concentrations ≥ 16.0 μmol/L, which is considered as an independent risk factor for birth defects, including CHDs [6, 7]. Since MTR is the only mammalian enzyme that metabolizes N^5^-MeTHF to THF and converts Hcy to Met, MTR activity is critically important for both folate and Hcy metabolism.

Noticeably, vitamin B12 (VB12 or cobalamin) is a key co-enzyme for MTR activity as VB12 deficiency invariably compromises MTR activity and results in elevated Hcy levels. Low VB12 status has also been postulated as a potential risk factor for intrauterine growth retardation and birth defects, including neural tube defects (NTDs) [8]. Dietary intake is the main source of circulating VB12. After intestinal absorption, VB12 binds to two types of transcobalamin proteins in plasma, including haptocorrin and transcobalamin-II (TCN2). Although TCN2 only carries a minor portion (˜20%) of the circulating VB12 [9, 10], TCN2-bound VB12 (also called holo-transcobalamin; holo-TC) is the only form for cell absorption via the endocytic receptors, such as the low density lipoprotein receptor-related protein-2 (LRP2) in kidneys, and CD320 in other cells of the body [11]. Therefore, holo-TC concentration is considered as an indicator of the status of VB12 in an individual [12–14].

Due to the importance of folate, Hcy and VB12 metabolism, functional SNPs in those critical genes have been screened in CHD patients in the past [15–17]. The variants in several genes involved in fetal absorption and transport of VB12 were found to be associated with increased risks for CHDs. For example, the intrinsic factor-cobalamin receptor CUBN is involved in the VB12 absorption in the terminal ileum. A genome-wide association (GWAS) study showed the rs11254363 A>G in the intron of *CUBN* is related to increasing VB12 levels [18]; and we have previously found that the rs11254363 G allele of *CUBN* significantly reduced the risk of CHDs [19]. However, no significant association of SNPs in *TCN2* and CHDs has been previously reported. For example, the rs1801198 (776C>G, Pro259Arg) is the most comprehensively studied polymorphism in *TCN2*. The effect of this polymorphism on VB12 concentrations contradicts several previous studies. It has been reported that 776 CC homozygotes have higher concentrations of total VB12, holo-TC, and lower Hcy levels than the other two genotypes [20–24], while other reports state that the 776 C>G polymorphism had no effect on VB12 levels [25, 26]. Also, no significant association of 776 C>G with CHDs was ever identified [27, 28].

In this study, a comprehensive list of 46 genes involved in folate metabolic pathway was studied in a northern Chinese population by target-sequencing. Among the 386 common polymorphisms (MAF > 5%) we identified, the c.230A>T (p. Lys77Met, rs75680863) of *TCN2* is the only missense polymorphism which is significantly associated with reduced CHD risk. The association was also consistently replicated in larger validation cohorts. Moreover, the c.230A>T is a specific polymorphism in East-Asian populations and exhibits nominally significant evidence for positive selection in Northern Chinese populations. We also observed that the concentration of holo-TC in the blood of women with the T/A genotype is higher than that in women with the A/A genotype, and functional experiments demonstrated that TCN2 77Met have a higher reabsorption rate than TCN2 77Lys.

## RESULTS

### c.230A>T (p.Lys77Met, rs75680863) in *TCN2* is associated with decreased risk of CHD in the population of Northern China

To detect any association of common variants in genes of the one carbon metabolism pathway to risk of CHDs, 46 candidate genes were target-captured sequenced in our cohort containing 412 CHD patients and 213 controls. We identified 386 common SNPs (MAF > 0.05) in those genes (Supplementary Table 4). Within them, sixteen SNPs in seven genes nominally differed between the CHDs and controls in an additive genetic model, including common variants in intron and 3UTR of *MTR*, 5′ upstream regions of *PRMT5* (protein arginine methyltransferase 5), and coding region of *TCN2* (Supplementary Table 4). No adjusted *p*-value was found to remain significant after Bonferroni adjustment. Notably, *TCN2* rs75680863 (c.230A>T) is the only missense variant associated with the risk of CHDs (*P* = 0.003 in additive model, Supplementary Table 4 and Table 1). We observed lower frequencies of the combined A/T and T/T genotypes in CHDs (37.5%) than controls (50.9%) (OR = 0.59, 95% CI = 0.39–0.89 in dominant model; OR = 0.38, 95% CI = 0.17–0.83 in recessive model; and OR = 0.61, 95% CI = 0.44–0.85, *P* = 0.003 in additive model) (Table 1). This suggested that there is a protective effect of the c.230T allele against CHDs. In addition, we did not observe any association between the previously reported *TCN2* rs1801198 (c.776G>C) and CHD risk in our cohort (Supplementary Table 4).

**Table 1:** Association of TCN2 c.230A>T allele with CHDs in two independent case–control studies

SNPs	Genotype^a^		MAF		Genetic model	OR (95% CI)	*P*-value^b^	*P*^c^, HWE test
	Control	Case	Control	Case				
First stage	104/90/18	256/132/21	0.30	0.21	Codominant	NA	0.001	0.87
					Dominant	0.59 (0.39–0.89)	0.012	
					Recessive	0.38 (0.17–0.83)	0.016	
					Additive	0.61 (0.44–0.85)	0.003	
Validation	662/426/89	237/157/18	0.26	0.23	Codominant	NA	0.003	0.08
					Dominant	0.75 (0.55–1.01)	0.057	
					Recessive	0.37 (1.20–0.67)	0.001	
					Additive	0.70 (0.55–0.89)	0.004	
Meta-analysis^d^	766/516/107	489/287/39	0.26	0.22	Codominant	NA	1.40e-05	0.13
					Dominant	0.69 (0.54–0.89)	0.003	
					Recessive	0.37 (0.23–0.60)	5.18e-05	
					Additive	0.67 (0.55- 0.81)	4.62e-05	

^a^AA/AT/TT; OR, odds ratio; CI, confidence interval; NA, not available; ^b^Adjusted by sex and age; ^c^HWE test in the controls; ^d^pooled values from fixed effects model (*P*_heterogeneity_ > 0.05).

To further confirm the relationship between *TCN2* c.230A>T and CHDs in the Northern Chinese population, we performed a validation study with enlarged samples that now included 412 CHDs and 1177 controls. Consistent results were obtained with this SNP (OR = 0.37, 95% CI = 0.20–0.67, *P* = 0.001 in recessive model; OR = 0.37, 95% CI = 0.55–0.89, *P* = 0.004 in additive model) (Table 1). Subsequently, we combined the samples and demonstrated that genotype distribution was nominally different between CHDs and control groups (*P* = 1.40e–05 in codominant model; *P* = 0.003 in dominant model; *P* = 5.18e-05 in recessive model). The CHD risk was decreased by 32% with the minor 230T allele (OR = 0.67, 95% Cl = 0.55–0.81, *P* = 4.62e–05 in additive model) compared to the 230A allele. The frequencies of all genotypes were in accordance with the Hardy-Weinberg expectation among control subjects (*P* > 0.05) (Table 1).

A further stratified analysis of 230A>T was performed based on different subtypes of CHDs [1]. The most significant difference in case *vs*. control was observed among septation defects (*P* = 0.0004 in additive model after adjusted by sex and age) (Table 2) and conotruncal defects (*P* = 0.014 in additive model after adjusted by sex and age). There is no such significant difference observed for 230A>T allele in other subtypes including right ventricular outflow tract obstruction (RVOTO) (*P* = 0.119 in additive model) and PDA (*P* = 0.25 in additive model) (Table 2).

**Table 2:** Stratification analysis of TCN2 c.230A>T according to CHD classification and phenotype

CHD Classification	Genotyping in Cases^a^	Genetic model	OR (95% CI)^b^	*P*-value^b^
Septation defects	251/142/17	Codominant	NA	0.001
		Dominant	0.67 (0.51–0.89)	0.005
		Recessive	0.37 (0.21–0.68)	0.001
		Additive	0.77 (0.64–0.93)	0.0004
Conotruncal defects	113/65/10	Codominant	NA	0.035
		Dominant	0.70 (0.48–1.02)	0.060
		Recessive	0.41 (0.19–0.87)	0.020
		Additive	0.69 (0.57–0.93)	0.014
RVOTO	36/21/3	Codominant	NA	0.290
		Dominant	0.70 (0.40–1.22)	0.206
		Recessive	0.44 (0.13–1.48)	0.185
		Additive	0.70 (0.45–1.10)	0.119
PDA	34/15/5	Codominant	NA	0.390
		Dominant	0.66 (0.37–1.21)	0.170
		Recessive	0.86 (0.32–2.32)	0.760
		Additive	0.77 (0.49–1.21)	0.250

^a^AA/AT/TT; ^b^Adjusted by sex and age.

### Distribution of *TCN2* c.230A>T in North Chinese and South Chinese

When we check this SNP *TCN2* rs75680863 (c.230A>T) in public database, we noticed that the T allele frequency was only 0.669% reported in dbSNP138 database, while it was 26% in our control group (Table 2). Based on the published data from the 1000 Genomes Project, the minor T allele was only observed in East-Asians, not in Europeans, Africans or Americans. The frequency of the T allele was greater in the Han Chinese in Beijing than that in Southern Han Chinese and Japanese in Tokyo, Japan (Table 3). Thus, the variant exhibits noticeable population specificity. These results indicated that there might be selection pressure present in the Han Chinese in Beijing population to maintain the higher frequency of the T allele. To evaluate whether positive selection was statistically significant, we analysed all SNP data of chromosome 22 from the 1000 Genome Project for Han Chinese in Beijing population by different statistical tests. According to *iHS* [29] and *rehh* [30] models, the derived allele T was positively selected on chromosome 22 among the Han Chinese in Beijing population (CHB), with an empirical *P* value at 0.023 and 0.032, respectively. SCCT [31], a statistical method used to pinpoint causal variants, also demonstrated that the derived allele T was positively selected on chromosome 22 in CHB population, with an empirical *P* value at 0.0086.

**Table 3:** Population diversity of the c.230A>T allele

Population		Frequency of T allele
East Asian	CHB	0.14
	CHS	0.05
	JPT	0.03
European	CEU	0
	FIN	0
	GBR	0
	IBS	0
African	ASW	0
	LWK	0
	YRI	0
Americas	MXL	0
	PUR	0
	CLM	0

*Data from 1000 Genomes; CHB: Han Chinese in Bejing, China; CHS: Southern Han Chinese, China; JPT: Japanese in Tokyo, Japan; CEU: Utah residents with Northern and Western European ancestry; FIN: Finnish in Finland; GBR: British in England and Scotland; IBS: Iberian populations in Spain; ASW: African Ancestry in Southwest US; LWK: Luhya in Webuye, Kenya; YRI: Yoruba in Ibadan, Nigeria; MXL: Mexican Ancestry in Los Angeles, California; PUR: Puerto Rican in Puerto Rico; CLM: Colombian in Medellin, Colombia.

### Association of *TCN2* c.230A>T with plasma holo-TC concentration in Chinese women

Since the c.230A>T allele may be under positive selection in the Northern Chinese population and statistically associated with the reduce risk of CHDs, we were interested in establishing whether the variants produced any functional changes in the TCN2 protein. The 77Lys (c.230A) residue located in the third alpha-helix of the TCN2 protein [32] and is highly conserved between species (Supplementary Figure 1A). The c.230T changes the amino acid residue from Lys to Met, which dramatically alters its chemical character, as lysine residues are hydrophilic and bear a positive electrical charge, whereas hydrophobic methionine residues do not have any charge.

**Figure 1:**
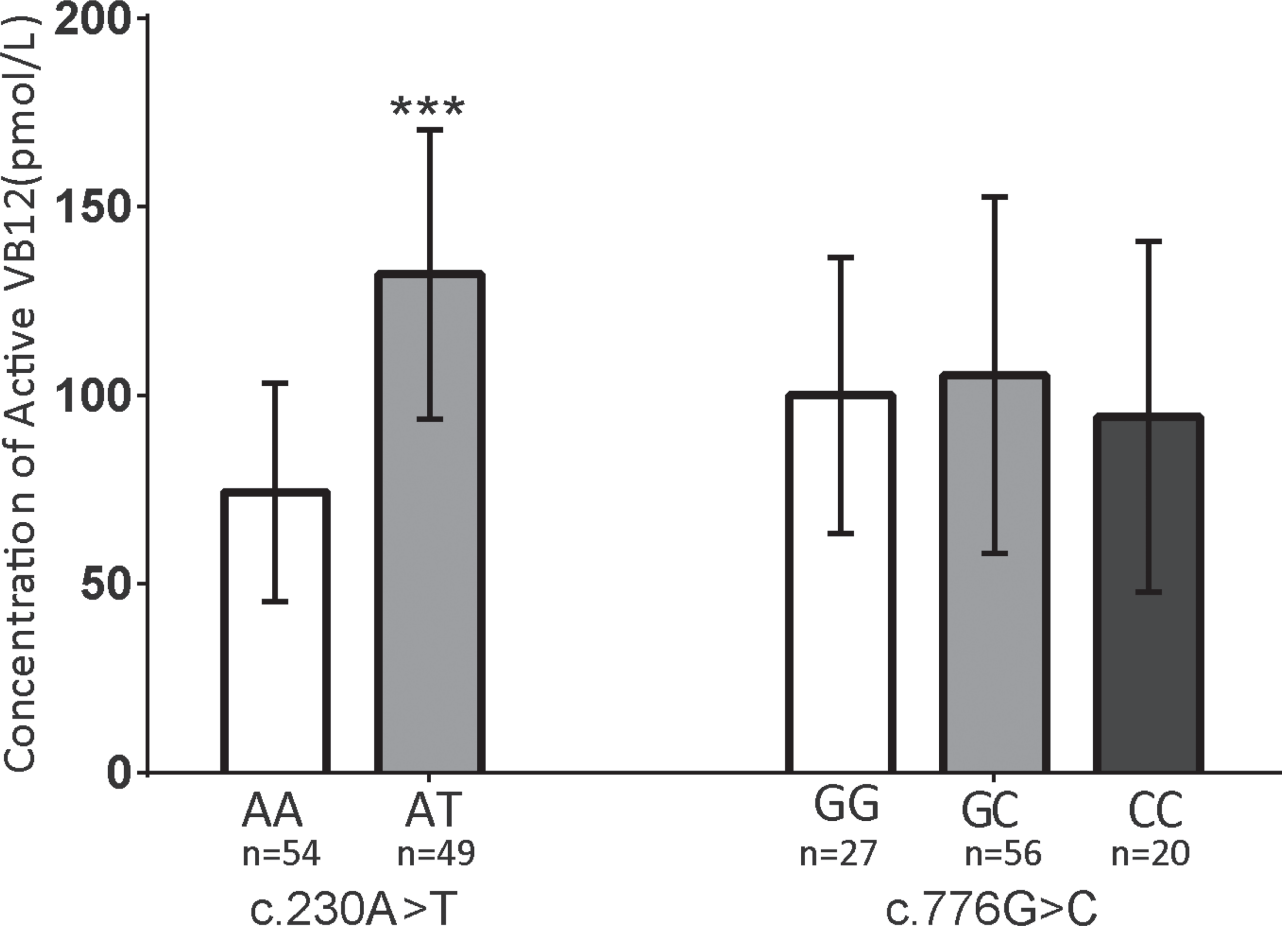
The c.230A>T allele correlates with the concentration of active VB12 (holo-TC) in human plasma The concentrations of holo-TC were measured within women with different genotypes. Plasma from AA:AT:TT = 54:49:0 individuals at c.230 and GG:GC:CC = 27:56:20 individuals at c.1025 were tested. The color coding used in the figure for each genotype is as follow, on the left side: white-AA, gray-AT; on the right side: white-GG, gray-GC, black-CC. The levels of holo-VB12 were significantly different between the individuals with different genotypes at c.230 A>T locus, but not at c.1025 G>C. Data are shown as mean ± SD. ***P < 0.001. (c.230 A>T: AA = 74.4 ± 29.0 pmol/L, AT = 132.1 ± 38.4 pmol/L; c.1025 G>C: GG = 100 ± 36.6 pmol/L, GC = 105.4 ± 47.3 pmol/L, CC = 94.4 ± 46.5 pmol/L).

To investigate the effect of this variant, we tested the holo-TC concentration in Chinese individuals with different genotypes at c.230 of *TCN2*. Ideally, we would prefer to test this correlation in the same Northern Chinese population in which we performed genotyping. Unfortunately, there were no such plasma samples available from the Northern China cohort. However, since the Southern China population frequency of the T allele is 5% (CHS, Table 3), we enrolled 487 healthy women with varied TCN2 genotypes from Shaoxing, a city in South China. Sanger sequencing of the c.230 loci was performed in these 487 women, of whom 49 were identified to have the A/T genotype at c.230, and the rest were A/A genotype. No T/T homozygous individuals were identified. The frequency of the minor T allele (5.03%) is consistent with the CHS data from the 1000 Genomes Project (Table 3). Plasma holo-TC concentrations in the 49 A/T heterozygotes and another 54 randomly selected individuals with the A/A genotype were determined. The holo-TC concentrations in the A/A women are 74.4 ± 29.0 pmol/L, which is similar to previously reported concentrations in the Vietnamese women (mean value 78 pmol/L) [33] and Chinese type 2 diabetes mellitus patients with VB12 > 400 ng/L (81.32 ± 32.91 pmol/L) [34]. However, the holo-TC concentrations were nearly twice as high in A/T heterozygotes (132.1 ± 38.4 pmol/L) compared with that of the A/A women (74.4 ± 29.0 pmol/L). Such correlation was not observed for the c.776 G>C variant (Figure 1). Our findings suggest that higher plasma holo-TC level is highly correlated with the c.230T allele, which was associated with a lower CHD risk.

### *TCN2* c.230A>T (p.Lys77Met) does not influence its ability to bind VB12

**Figure 2:**
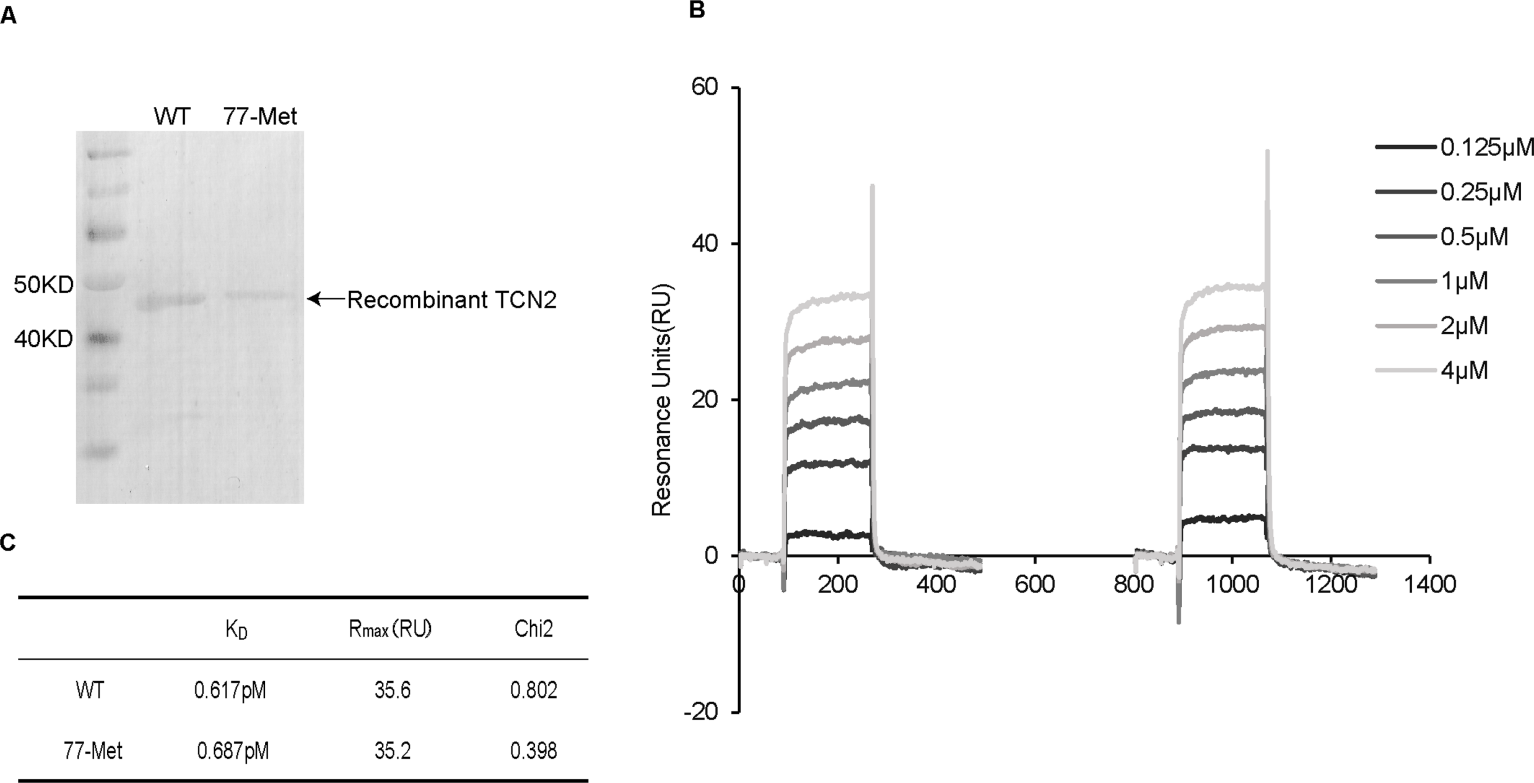
SPR analyzed the interaction between VB12 and recombinant TCN2 proteins (**A**) Purified recombinant TCN2 proteins were analyzed by using Tris–glycine–SDS followed by Coomassie brilliant blue G–250 staining. The molecular weights of recombinant TCN2 proteins were same as the predicted molecular weight (43 kDa). (**B**) Kinetic sensorgrams of TCN2-wild and 77-Met with different concentrations of VB12 (0.125–4 μM). (**C**) Dissociation Constants (KD) of wild and 77-Met proteins for binding of VB12.

There are several possibilities as to how the Lys77Met variant is associated with elevated plasma holo-TC levels in T allele heterozygotes. One possibility is due to an increased binding affinity of TCN2 to VB12. To test this hypothesis, SPR assay was performed to compare the binding ability to VB12 between the wild-type (WT) 77Lys and mutant 77Met TCN2. Initially, the recombinant 77Lys and 77Met TCN2 proteins were purified (Figure 2A) and immobilized on the sensor chip. Then, VB12 (0.125–4 μM) was applied to the sensor chip in a dose-dependent manner (Figure 2B). There was no significant difference on the dissociation constant (K_D_) between 77Lys (K_D_ = 0.617pM) and 77Met (K_D_ = 0.687pM) to VB12 (Figure 2C), suggesting that the c.230A>T mutation did not affect the binding ability of TCN2 to VB12.

### *TCN2* c.230A>T (p.Lys77Met) affects the distribution of holo-TC in and out of HEK293T cells through the LRP2 receptor

**Figure 3:**
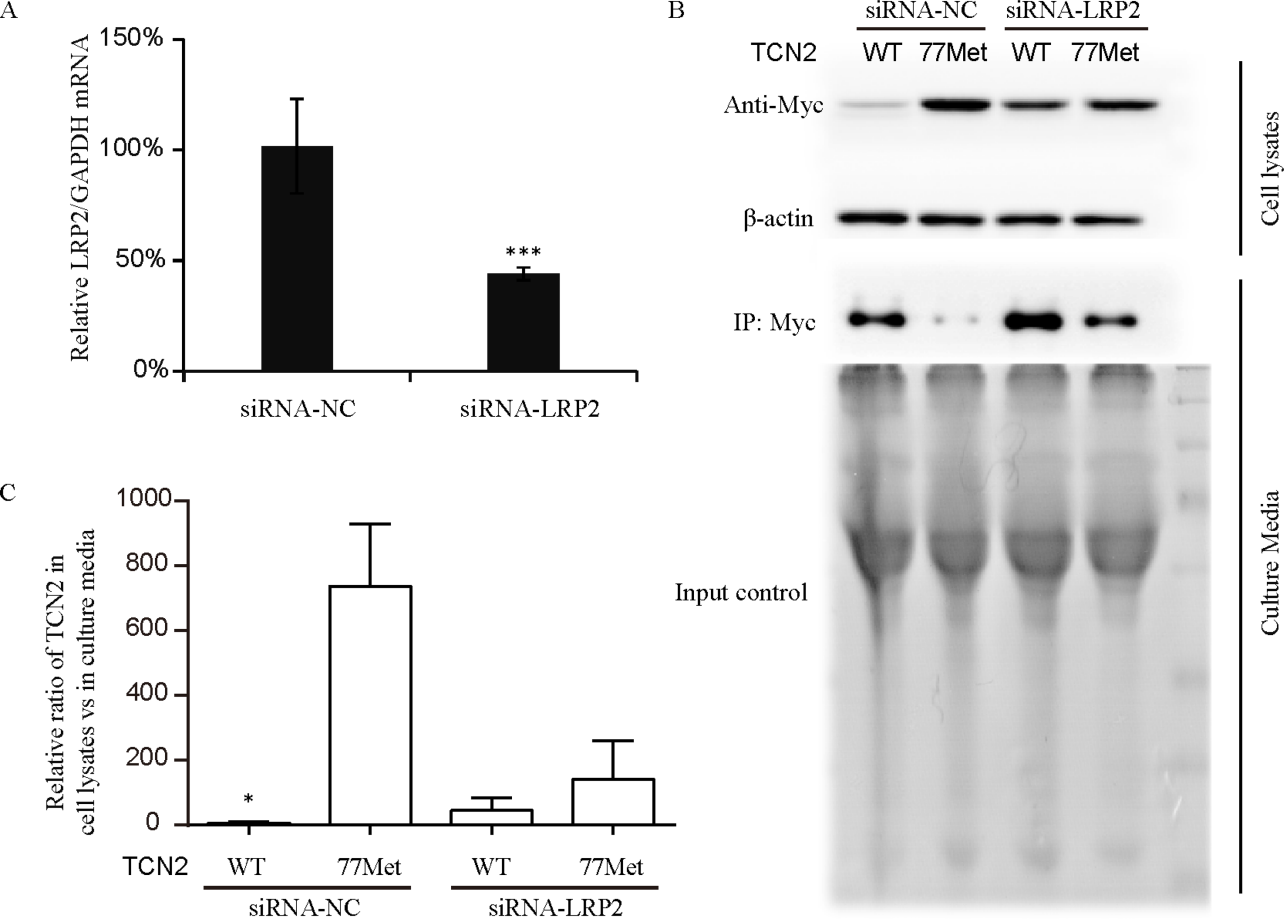
Quantification of TCN2-77Met and TCN2-77Lys (wild type, WT) protein in HEK293T cells (**A**) The knock-down efficiency of LRP specific siRNA was measured by detecting the mRNA level of LRP2, which was decreased more about 50% by the siRNA. HEK293T cells were transfected with 100 nM either negative control siRNA (siRNA-NC) or LRP2 siRNA. After 48 h, real-time-PCR analysis was carried out using specific primers for LRP2 and GAPDH. (**B**) and (**C**) HEK293T cells were transfected with myc-tagged TCN2-77Lys (wild type, WT) or TCN2-77Met expression plasmids. After 48 hrs, both cell lysate and culture media were collected for further Western blotting analysis. The band intensity of TCN2 WT and 77 Met proteins were normalized to individual β-actin in Western blotting or loading input in immunoprecipitation using Image-Pro Plus software 6.0. Cell lysates/culture media ratio of TCN2-WT was significantly lower than that of 77Met protein (*P* < 0.05) in siRNA negative control (siRNA-NC) group. However, after the LRP2 was knocked down, there was no difference between them. The results were repeated for three times.

Another possibility for the increased plasma holo-TC concentration is an increased rate of holo-TC reabsorption in the kidney in A/T heterozygous individuals. The holo-TC reabsorption in the kidney is specifically mediated by its high affinity receptor (LRP2) [35, 36], also known as Megalin, which is located on the apical membrane of proximal tubule cells [37]. The structure data of TCN2 showed that the α3 helix where p.Lys77Met located may belong to receptor-recognition region [32], and adjacent to hypothesized functional receptor binding sites [38]. Therefore, we hypothesized that the p.Lys77Met variant might influence the interaction between holo-TC and the LRP2 receptor. To test this hypothesis, human kidney epithelia HEK293T cells were used to overexpress either myc-tagged WT (77Lys) or myc-tagged 77Met TCN2 proteins and the exogenous TCN2-WT and 77Met proteins were evaluated in both culture-media and cell lysates. The results showed that most of TCN2-77Met protein was in cell lysates, and most of the TCN2-WT protein was in the media (Figure 3B and 3C). However, after siRNA knockdown of LRP2 (the knockdown efficiency was shown in Figure 3A), the protein levels of both TCN2-WT and 77Met in the culture media were increased (Figure 3B). The ratios of TCN2-WT in cell lysates to culture media was significantly lower than that of TCN2-77Met in the presence of LRP2; but the difference disappeared after LRP2 was knocked down (Figure 3C). Those results indicated that the TCN2-77Met could promote the reabsorption of holo-TC through LRP2 into cells.

## DISCUSSION

VB12 is critical for normal fetal development. In addition to CHDs, VB12 deficiency has been shown to contribute to the risk of several other birth defects, including NTDs [39] and orofacial clefts [40]. In the present study, we screened the common SNPs in 46 genes involved in folate and one carbon metabolism in our CHD cohort, and only one functional non-synonymous polymorphism c.230A>T in the *TCN2* gene was significantly associated with the risk for CHDs. It was demonstrated that the T allele significantly reduces the risk of CHDs. Other associated sites we identified include common variants in 3′UTR of *MTR* and 5′ upstream regions of *PRMT5* (a protein involved in the histone methylation [41]). These results correlate with our previous results that genetic variants of MTR regulatory regions [42] and rare mutations in histone modification genes are related to the pathogenesis of CHDs [43, 44].

It has been shown that the genetic causes of each specific subtype of CHDs also have high degree of heterogeneity, and different subgroups of CHDs can result from the same chromosomal alteration or mutations in the same gene. For example, trisomy 21 may cause atrioventricular, Tetralogy of Fallot (TOF), or patent ductus arteriosus (PDA). Deletion 22q11 may cause TOF as well as interrupted aortic arch [45]. Therefore, we analyzed the association of *TCN2* c.230A>T to CHDs collectively as well as separately among several major subtypes of CHDs. When analyzing the association with specific types of CHDs, we observed that the TCN2 230T allele significantly reduced the risk of septation and conotruncal defects, but not RVOTO or PDA (Table 2). Insufficient sample size in our study may result in failing to find positive associations being replicated in RVOTO and PDA. However, several large population-based case–control studies also reported the association between periconceptional multivitamins supplementation and the reduced risk for septation and conotruncal defects [46–48]. The specific mechanisms underlying the protective effects of folate and VB12 on septation and conotruncal defects remain to be elucidated.

Both maternal and fetal vitamin metabolisms need to be considered as the homeostasis of VB12 metabolism during fetal development is affected by both maternal and fetal genetic susceptibilities [49, 50]. Before and during pregnancy, maternal folate and VB12 status are closely associated with fetal development, and poor maternal VB12 status could impact fetal DNA methylation reactions and increase the risk of gestational diabetes, early miscarriage and congenital defects [51–53]. Herein, we found that the *TCN2* c.230T (p.77Met) is significantly associated with elevated plasma holo-TC in women. To investigate the possibilities behind this elevated plasma holo-TC in women with 77Met TCN2, we performed both SPR and cellular expression experiments *in vitro*. It is not surprising that p.Lys77Met does not affect its binding to VB12, as this mutation site does not locate in the VB12 binding region. However, we found p.Lys77Met could influence the distribution of TCN2 protein inside or outside of the HEK293 cells. One possibility is due to different secretion efficiency of those two proteins. However, p.Lys77Met is not located in the signal peptide, which is at the N-terminal 1–18 amino acids. Therefore, it is unlikely the different levels of TCN2 between inside and outside of HEK293 cells are due to secretion deficiency of TCN2 77Met protein. Another possibility for the distribution difference is due to different degradation efficiency of the 77Lys and 77Met TCN2. However, the most abundantly ubiquitylated Lys residues which signal protein degradation are Lys48 and Lys63, and even some atypical ubiquitylation sites do not include Lys77 [54]. Therefore, it is unlikely Lys77Met affects TCN2 degradation. However, further analysis is needed to fully address this issue. The last possibility is due to different reabsorption of TCN2 due to its affinity change to its receptor. This hypothesis is strongly supported by both the mutation location, which is in suggested receptor-recognition region [32, 38], and our results after LRP2 knockdown (Figure 3). As the LRP2 receptor is involved in holo-TC endocytosis in epithelial cells, especially in the renal proximal tubule cells [55], it is likely the elevated plasma level of holo-TC in women with *TCN2* 230T allele may be due to LRP2 facilitated reabsorption in kidney.

Noticeably, LPR2 is also expressed in the embryo and endoderm of the visceral yolk sac and in the placental syncytiotrophoblast cells [56]. Therefore, not only the *TCN2* 230T allele in mother's side could elevate plasma holo-TC, but also babies with *TCN2* 230T allele could be further benefited as probably more VB12 is transported across the yolk sac and placenta into the developing fetus. Since the maternal and fetal genes could interact each other to influence the phenotypes of mothers and babies [57], it is necessary to investigate the interaction of c.230A>T in the mother and offspring and other non-genetic factors in association with CHD risk in the future. However, the transporting processes of maternal-embryonic VB12 are still poorly understood. Besides LRP2, other receptors, such as CD320, which is also expressed in placental tissue [58], may be also involved in maternal-embryonic VB12 transporting. Further investigations are needed to explore the interaction between the TCN2 variants and CD320 to understand the detailed mechanisms of maternal-embryonic VB12 transporting.

We observed that the frequency of this putatively protective allele 230T in Northern Chinese populations is much higher than that of Southern Chinese or other global populations (Table 3). It is noticed that the MAF (0.25) of the 230T allele in this study is higher than CHB (0.15) in 1000 Genome Project. Such difference may be due to the mixed population in CHB (Beijing), as the residents in large cities originally came from different areas within China. The principal components (PC) analysis revealed that the CHB individuals are distributed widely in the Northern Han cluster (54.8%), the central Han cluster (13.1%), and the Southern Han cluster (32.1%) [59]. The southern Han population mixed in CHB population probably contribute to the lower MAF of TCN2 c.230T in CHB.

It has been reported that the functional genetic variants in key genes of the one carbon metabolic pathway vary across different geographical regions and ethnic groups. For example, the 5,10-methylenetetrahydrofolate reductase (MTHFR) gene polymorphism c.677C>T results in a decreased activity of the enzyme that is associated with multiple disease endpoints including birth defects [60]. The distribution of the 677T polymorphism among populations may be influenced by historic dietary folate availability [61] or environmental factors, such as ultraviolet (UV) radiation exposure [62]. A similar effect on the *TCN2* 230T allele might also exist in the north of China. Compared with the southerners in China, the northerners had higher prevalence of folate/VB12/VB6 deficiency and hyperhomocysteinemia [63], which is associated with the relatively high risk for birth defects, such as NTDs, in north of China. And the women in the northern China with periconceptional folic acid supplementation showed greater reduction in preventing the birth of infants with NTDs than women in the southern China [64]. Since VB12 is critical for fetal development, we report the highest observed population-specific frequency of 230T in a northern Chinese population, especially pregnant women to maintain high concentrations of VB12 and folate in blood. As we showed in our study, the concentration of active VB12 (holo-TC) in individuals with the 230T allele was higher than in those with the 230A allele. Therefore, individuals with *TCN2* 230T allele might have an advantage in maintaining higher active VB12 concentrations circulating in the blood, which in turn could promote the activity of MTR. Since MTR activity is critical for both folate and Hcy metabolism, the higher activity of MTR as a consequence of the *TCN2* 230T allele could be beneficial to early embryonic development and thereby reduce the risk of CHDs. This functional advantage could help to promote the spread of the 230T allele in northern China. Further population based studies and evolutionary analyses are needed to understand the influence on the distribution of TCN2 c.230 A>T in China.

In summary, we identified a novel functional variant in *TCN2* among patients with sporadic CHDs. The 230T allele was nominally associated with lower risk of CHDs in a Northern Chinese population. We also found the frequency of 230 T allele is higher in the Northern Chinese population than other populations, and it is also associated with higher concentrations of holo-TC compared to the 230A allele. Functional studies suggested that the 230T allele elevated the level of transcobalamin protein in HEK293T cells via the LRP2 receptor. Since the LRP2 receptor is expressed in both the renal proximal tubule cells and placental syncytiotrophoblast cells [55, 65], it is likely to facilitate more hole-TC with TCN2 77Met reabsorption in the kidney and transportation between the mother and fetus. Our results provided the evidence of an unexpected protective role of the TCN2 c230T allele against CHDs, and further support the importance of interrogating genetic variants that affect VB12 metabolism in association of CHDs. Such investigations will enhance our understanding of the interaction between nutrition and genetics, and their synergic effects with respect to birth defect.

## MATERIALS AND METHODS

### Study participants

The initial sequencing stage included 412 sporadic CHD patients recruited from the Cardiovascular Disease Institute of Jinan Military Command (Jinan, Shandong, China) between 2008 and 2009. For the validation stage, an additional 412 cases with CHDs were collected from the same hospital between March 2010 and March 2012. Sporadic CHD cases were diagnosed on the basis of echocardiography, with some diagnoses further confirmed by surgery. Patients who had additional clinical features including developmental anomalies were excluded from the study. Patients were also excluded if they had a positive family history of CHDs in a first-degree relative, maternal diabetes mellitus, maternal exposure to known teratogens or therapeutic drugs during pregnancy. All of the CHD cases were classified according to methods previously described by our laboratory [1] (detailed diagnosis information on the patients is provided in Supplementary Table 1). The 212 controls in first stage and the 1177 controls in the validation stage were all from Northern Han Chinese population and they are gender-matched, unrelated healthy volunteers recruited from the same geographical area (both *P* values in stage 1 and validation stage were *P* = 0.10, Supplementary Table 1). Controls with congenital anomalies or cardiac disease were excluded.

The experiments were conducted in accordance with the Declaration of Helsinki. Protocols were reviewed and approved by the Ethics Committee of the School of Life Sciences, Fudan University prior to the commencement of the study. Written informed consent from the parents or guardians of all children was obtained.

### DNA sequencing and genotyping

The 5′-UTR, 3′-UTR, and coding regions in 46 genes of folate and one carbon metabolism were selected to be sequenced by next generation sequencing (Supplementary Table 2). The genomic structures of candidate genes were determined using human genome assembly GRCh37/hg19. Genomic DNA-fragment libraries and target enrichment using specific probes for target genes were performed using the Agilent SureSelect XT Custom Enrichment System with slight modifications, as previously described [66]. In brief, 1ug of genomic DNA was subjected to ultrasonic energy fragmentation and then ligated with an indexed adapter. Each of the 48 individual libraries were equivalently pooled and then hybridized to RNA library baits. Sequencing was performed on an Illumina HiSeq2000 DNA sequencer (version 3, Illumina, Inc.). Variants were called using SNPTools [67], and the variants with a quality score below 1.5 were eliminated. Variants were annotated with gene function data from UCSC and known variants were assigned respective SNP codes as dbSNP137. The SNPs with high missing genotype rates (less than 90%) and deviating from Hardy-Weinberg equilibrium in the controls (*P* < 0.05) were also excluded. Finally, 386 SNPs found in one carbon metabolism genes were further analyzed. To confirm the next generation sequencing genotyping results, about 25% of the samples were randomly selected to perform Sanger sequencing on rs75680863 and rs1801198, and the results were 99.01% concordant. The primers for the PCR and the DNA sequencing are provided in Supplementary Table 3.

For the validation stage, we designed primers to PCR-amplify and sequence the DNA fragment containing rs75680863 in all of the samples (412 CHDs + 1177 Controls). About 10% of the samples (160) were randomly selected to be genotyped again by Sanger sequencing, and the results were 100% concordant.

### Plasma holo-TC detection

For the holo-TC detection, blood samples of 487 healthy women from Shaoxing (age: 29 ± 5.2) were collected. Genotyping on both rs75680863 and rs1801198 were performed using Sanger sequencing. 49 women with the A/T genotype of rs75680863 were identified and no T/T individuals on this site were identified amongst the 487 healthy controls. For rs1801198, 125 women (25.7%) had the C/C genotype, 274 women (56.3%) had the C/G genotype, and 88 women (18.1%) had the G/G genotype. These 49 heterozygous individuals for rs75680863 and an additional 54 randomly selected individuals with the A/A genotype were tested for their plasma holo-TC concentrations. The active VB12 (holotranscobalamin) Enzyme Immunoassay (EIA) Kit (Axis-Shield, Scotland) was used to determine plasma holo-TC levels, according to the manufacturer's instructions. The assays were performed by laboratory personnel who were blinded to subject genotype. Each test was performed in duplicate, and the mean level was used for further analysis. The standard samples in the kit were used to assess the precision in different runs. The mean values of intra-assay and inter-assay coefficient of variation (CV) was 6.2% and 8.5%. The mean CV value in rs75680863 AT groups is 3.7%, and 6.9% for AA groups.

### Plasmid construction and site-directed mutagenesis

Human *TCN2* cDNA was purchased from Sino Biological Inc. (Beijing, China). The *TCN2* mutant was generated by the Quick Change^®^ Site-Directed Mutagenesis Kit (Stratagene). The wildtype and mutant ORF of *TCN2* were cloned into pcDNA^™^3.1/Myc-His (−) B vector (Invitrogen) at *XhoI* and *KpnI* restriction sites for protein purification, Western blotting, and immunoprecipitation (IP) studies. The primers used for these studies are provided in the Supplementary Table 2. All of the plasmids were verified by direct double-strand DNA sequencing prior to use.

### Purification of the recombinant human TCN2

Human HEK293T cells were seeded in a 10-cm^2^ dish and allowed to grow to 70–80% confluence. The cells were subsequently transfected with 10μg of plasmid either expressing TCN2-230A (77Lys) or TCN2-230T (77Met) by using Lipofectamine2000 (Invitrogen, Shanghai, China). The transfected cells were cultured in DEME high glucose media (Gibico, Shanghai, China) without serum. 24 hours post-transfection, approximately 10 ml of media was harvested and centrifuged at 4°C to remove cell debris. The supernatant was loaded into SnakeSkin Dialysis Tubing (MWCO 10 K, Thermo, China) and dialyzed in imidazole binding buffer (50 mM Tris-HCl, pH 8.0, 300 mM NaCl, 10 mM imidazole) at 4°C until the supernatant turned to a light yellow color. The supernatant was purified by Ni-NTA chromatography (1 ml Ni-NTA Superflow Cartridge, Qiagen, Shanghai, China) according to the manufacturer's protocol. After washing with wash-buffer (50 mM Tris-HCl, pH 8.0, 300 mM NaCl, 20 mM imidazole), the recombinant protein was eluted using elution buffer (50 mM Tris-HCl, pH 8.0, 300 mM NaCl, 110 mM imidazole). Further purification was achieved by a gel filtration step (Superdex 75 HR 10/300 GL, GE-Healthcare, USA) using an AKTA FPLC system (GE Healthcare). The protein was analyzed by 10% SDS–PAGE and concentrated in an Amicon Ultra-2 Centrifugal Filter Unit (MWCO 10 K, Millipore, Shanghai, China). Finally, purified protein dissolved in PBS (pH 7.0, 30% Glycerin) was concentrated to approximately 50 μg/ml and stored in aliquots at −80°C. The purified proteins were examined on a standard Tris–glycine–SDS PAGE followed by Coomassie brilliant blue G–250 staining.

### Surface plasmon resonance (SPR) analysis

The interaction between TCN2 and VB12 was performed on a BIAcore 3000 instrument (GE Healthcare Life Sciences, Ohio, USA). The PBS buffer (pH 7.0) was used in the Biacore experiments on nitrilotriacetic (NTA) chips. Briefly, the surface of the NTA chip was activated by Ni^2+^ according to the suppliers’ recommendation. Subsequently, His-tagged TCN2 recombinant proteins (wild-type and mutant, 7.5 μg/ml) were resuspended in PBS and bound to flow cell 2 (FC2) and FC3 on a Ni-NTA chip prepared according to instructions from Biacore. The FC1 (no binding of VB12 occurred) was used as reference cell. The binding assays were performed in reaction buffer (PBS, pH 7.0) at 25°C. Varying concentrations of cyanocobalamin (VB12, V2876, Sigma, Shanghai, China) were passed over the FC1, FC2 and FC3 of the sensor chip at a flow rate of 20 μl/min for 3 min. The sensorgrams were normalized relative to the highest R_max_, expressed as relative units (RU) and analyzed with BIAevaluation 4.1 software using a global fitting procedure and various kinetic models.

### LRP2 knockdown using siRNA and quantitative PCR (qPCR)

The sequence of human LRP2 used in the siRNA studies were: sense 5′GCAGCUUACUUGUGACAAU3′ and antisense 5′AUUGUCACAAGUAA GCUGC3′, which were synthesized and purified by RiboBio Co., Ltd. Scrambled siRNA (Cat. no: siN05815122147, RiboBio, Guangzhou, China) was used as a control for comparison. 6 × 10^5^ HEK293T cells were seeded in 6-well plates and transfected with 100 nM siRNA and 400 ng 77Lys/77Met TCN2 plasmid. After 48 h, total RNA was extracted from the transfected cells using the miRNeasy Mini Kit (Qiagen, Beijing, China) and was converted to cDNA using random hexamers, oligo (dT) primers and Moloney murine leukaemia virus reverse transcriptase (TaKaRa, Dalian, China). *LRP2* mRNA levels were measured using qPCR with the StepOnePlus system (ABI, Foster City, CA). These studies utilized *GAPDH* as an internal reference gene. The reaction mixture contained 10μM of each primer, 2× SYBR Green PCR Master Mix (ABI, CA, USA) and 1 μl cDNA. The primers used are listed in Supplementary Table 2. Each reaction was performed in triplicate.

### Immunoprecipitation and Western blot

At 48 hrs post-transfection with LRP2-siRNA and TCN2 77Lys/77Met plasmids, whole-cell lysates in 6-well plates were washed with cold PBS and harvested in RIPA buffer (Sigma) containing a protease inhibitor cocktail (Roche, Indianapolis, IN, USA). The approximately 3 mls of media were concentrated to 100 μl using centrifugal filters for collection of proteins larger than 10 kDa (Millipore, Billerica, MA). An aliquot of concentrated supernatants (500 μg protein) were incubated with 10μl of anti-c-Myc agarose slurry from Pierce c-Myc Tag IP/Co-IP kit (Thermo Scientific), followed by gentle end-over-end mixing at 4°C overnight. The agarose samples were washed 3 times using TBS plus 0.05% Tween-20 and then eluted using non-reducing buffer. The eluted proteins were subjected to Western blot analysis. Lysate proteins (50 mg) were separated on 12% glycine SDS-PAGE gel and transferred to a PVDF membrane. Membranes were blocked in 5% dry milk in TBS with 0.1% Tween-20 for 1h at room temperature followed by incubation with Anti-c-Myc (1:1000 dilution, Sigma Aldrich) and Anti-β-actin (1:3000 dilution, Sigma Aldrich). Immunoblot signals were visualized with Super Signal West Pico chemiluminescent substrate (Pierce Biotechnology). The quantification of band intensity was carried out using Image-Pro Plus software 6.0. Band intensity of TCN2 77Lys and 77Met proteins were respectively normalized to individual β-actin in Western blotting or loading input in immunoprecipitation. The ratio of proteins in the cell lysates and culture media were compared between TCN2 77Lys and 77Met.

### Statistical analysis

The analysis of the potential of positive selection for *TCN2* c.230A>T in the Han Chinese in Beijing China [CHB] population (http://www.1000genomes.org), three methods were used. The first two are based on the extended haplotype homozygosity (*EHH*) test. We used the test statistic of integrated EHH (*iHS*) [29] and *rehh*, which is a software R package for *EHH*-based tests [30]. The third method is the selection by conditional coalescent tree (*SCCT*) [31]. As all three methods are used to calculate the probability of positive selection within a given surrounding chromosome region, therefore *TCN2* c.230A>T is compared to all common SNPs (MAF ≥ 0.05) of chromosome 22 in CHB population. The *EHH* method is to measure to which extent an extended haplotype is transmitted without recombination. If the core allele is under selection, then the *EHH* value is expected to be close to 1 over a large distance upstream and downstream the focal SNP. The high values of *EHH* (i.e. ˜1) and a high population frequency indicate the core allele rose faster than expected under neutral evolution. *iHH* is an empirical test based on the integral of the observed decay of *EHH* and *iHS* is the log-ration of *iHH* computed at the derived and the ancestral focal SNP alleles. The *SCCT* method is based on conditional coalescent tree, which take the imbalance of genetic variants near a potential candidate into consideration. The *SCCT* score for every common SNP of chromosome 22 in CHB population was computed. Then we partitioned SNPs into different bins, and normalize the score of SNPs by the mean and variance for each bin.

The Hardy-Weinberg equilibrium (HWE) was tested using the χ^2^ test for the controls. Odds ratios (ORs) and 95% confidence intervals (CI) were calculated using unconditional logistic regression analysis with adjustment for sex. The common SNPs with high missing genotype rates (less than 90%) and deviating from HWE in the controls (*P* < 0.05) were excluded. The analyses of the association between different genotypes and CHD risk were performed using the Plink1.9 software. All statistical tests were two-tailed with *P* < 0.05 set as the significance level.

## SUPPLEMENTARY MATERIALS FIGURES AND TABLES






