# Supplementary material for: A missense mutation in TCN2 is associated with decreased risk for congenital heart defects and may increase cellular uptake of vitamin B12 via Megalin
Source: Oncotarget. 2017 Jul 19;8(33):55216–29. doi: 10.18632/oncotarget.19377 (PMC5589654; doi:10.18632/oncotarget.19377)
Supplement: Supplementary file 1 [file oncotarget-08-55216-s001.pdf]

A missense mutation in *TCN2* is associated with decreased risk for congenital heart defects and may increase cellular uptake of vitamin B12 via Megalin

SUPPLEMENTARY MATERIALS

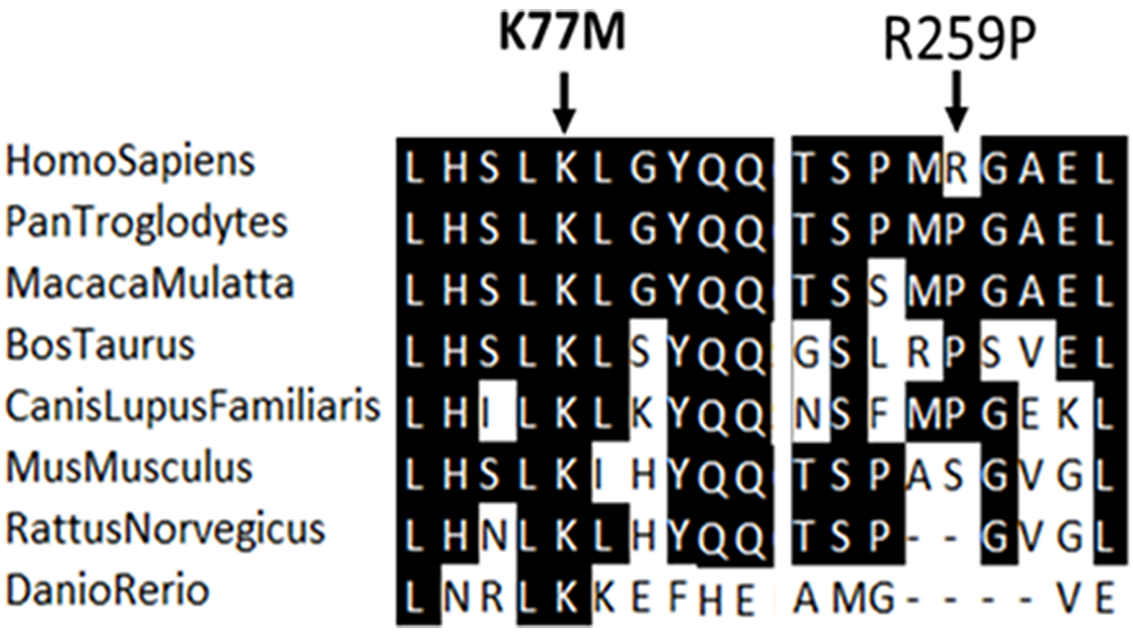

Supplementary Figure 1: Alignment of TCN2 ortholog protein sequences using the ClustalW method.

Supplementary Table 1: The detail information of in CHD cases and controls

Variable	Cases (%)	Controls (%)	*P* value
Stage 1, Sequencing Group	412	213	
Age, years (mean ± SD)	2.9 ± 2.5	7.2 ± 3.7	$P < 0.01$
Gender			
Male	229 (55.6%)	106 (49.8%)	0.10
Female	183 (44.4%)	107 (50.2%)	
Stage 2, Validation Group	412	1177	
Age, years (mean ± SD)	4.4 ± 5.2	27.7 ± 15.1	$P < 0.01$
Gender			
Male	201 (48.8%)	630 (53.5%)	0.10
Female	211 (51.2%)	547 (46.5%)	
**CHM classification**	**Pooling sequenceing**	**Validation**	**All**
Septation defects	137 (33.3%)	275 (66.7%)	412 (50.0%)
Conotruncal defects	140 (34.0%)	48 (11.7%)	188 (22.8%)
RVOTO	43 (10.4%)	18 (4.4%)	61 (7.4%)
PDA	17 (4.1%)	37 (9.0%)	54 (6.6%)
LVOTO	18 (4.4%)	13 (3.2%)	31 (3.8%)
AVSD	20 (4.9%)	7 (1.7%)	27 (3.3%)
APVR	11 (2.7%)	7 (1.7%)	18 (2.2%)
Complex	10 (2.4%)	2 (0.5%)	12 (1.5%)
Heterotaxy	3 (0.7%)	2 (0.5%)	5 (0.6%)
Others	13 (3.2%)	3 (0.7%)	16 (1.9%)

RVOTO, Right Ventricular Outflow Tract Obstruction; PDA, patent ductus arteriosus; LVOTO, Left Ventricular Outflow Tract Obstruction; AVSD, Atrioventricular Septal Defect; APVR, Anomalous pulmonary venous return.

Supplementary Table 2: Candidate Genes Selected for Association Study of Common Genetic Variation with CHD. See Supplementary_Table_2

Supplementary Table 3: DNA/RNA sequence of all used primer pairs

Primer Name	Sequence (5′–3′)	Purpose
rs75680863-F	CTCCTCCCACTGCCTTTCAG	PCR/Sequence
rs75680863-R	AAAGTGGTGACAGGCCCAAA	
rs1801198-F	GGTGCTGGAACACCTAGCC	PCR/Sequence
rs1801198-R	GCTCCATCCTGCAGACTGG	
Plasmid-F	CCGCTCGAGATGAGGCACCTTGGGGCC	Construct
Plasmid-R	CGGGGTACCCAGCTAACCAGCCTCAGCTCA	
Mutation-F	CACAGCCTCATGCTTGGTTAC	Point mutation
Mutation-R	GTAACCAAGCATGAGGCTGTG	
LRP2-F	GTTCAGATGACGCGGATGAAA	qPCR
LRP2-R	TCACAGTCTTGATCTTGGTCACA	
GAPDH-F	GAAACTGTGGCGTGATGGC	qPCR
GAPDH-R	CACCACTGACACGTTGGCAG	
LRP2 siRNA sense	GCAGCUUACUUGUGACAAU	knockdown
LRP2 siRNA antisense	AUUGUCACAAGUAAGCUGC	knockdown

Suppplementary Table 4: Association of common SNPs in folate-related genes with CHDs in the population of Northern China. See Supplementary_Table_4
