# Supplementary material for: A missense mutation in TCN2 is associated with decreased risk for congenital heart defects and may increase cellular uptake of vitamin B12 via Megalin
Source: Oncotarget. 2017 Jul 19;8(33):55216–29. doi: 10.18632/oncotarget.19377 (PMC5589654; doi:10.18632/oncotarget.19377)
Supplement: Supplementary file 2 [file oncotarget-08-55216-s002.docx]

**Supplementary Table 2. Candidate Genes Selected for Association Study of Common Genetic Variation with CHD**

| **Gene** | **Description** |
| --- | --- |
| AHCY | S-Adenosylhomocysteine hydrolase­ |
| ALDH1L1 | Aldehyde dehydrogenase 1 family, member L1 |
| AMD1 | Adenosylmethionine Decarboxylase 1 |
| BHMT | Betaine-homocysteine methyltransferase1 |
| BHMT2 | Betaine-homocysteine methyltransferase2 |
| CBS | Cystathionine-Beta-Synthase |
| CHKA | Choline Kinase Alpha |
| CTH | Cystathionase (cystathionine gamma-lyase) |
| DHFR | Dihydrofolate reductase |
| DNMT1 | DNA cytosine 5 methyltransferase1 |
| DNMT3A | DNA cytosine 5 methyltransferase3a |
| DNMT3B | DNA cytosine 5 methyltransferase3b |
| FOLH1 | Folylpolyglutamate hydrolase (gamma-glutamyl hydrolase, Glutamate carboxypeptidase II) |
| FOLR1 | Folate receptor alpha |
| FOLR2 | Folate receptor 2 (fetal) |
| FOLR3 | Folate binding protein gamma |
| FPGS | Folylpolyglutamate synthase |
| FTCD | Formiminoglutamate formiminotransferase-cyclodeaminase |
| GAMT | Guanidinoacetate N-Methyltransferase |
| GART | Phosphoribosylglycinamide formyltransferase/synthetase, phosphoribosylaminoimidazole synthetase |
| GGH | Lysosomal gamma-glutamylhydrolase |
| GNMT | Glycine N-methyltransferase |
| MAT1A | Methionine adenosyltransferase 1 |

**Supplementary Table 2. Candidate Genes Selected for Association Study of Common Genetic Variation with CHD** *(Continued)*

| **Gene** | **Description** |
| --- | --- |
| MAT2A | Methionine adenosyltransferase II alpha |
| MGMT | O-6-Methylguanine-DNA Methyltransferase |
| MTHFD1 | Methylenetetrahydrofolate dehydrogenase (NADP + dependent) 1, methenyltetrahydrofolate cyclohydrolase, formyltetrahydrofolate synthetase (cytosolic) |
| MTHFD2 | Methylenetetrahydrofolate dehydrogenase (NADP + dependent) 2, methenyltetrahydrofolate cyclohydrolase (mitochondrial) |
| MTHFR | Methylenetetrahydrofolate reductase |
| MTHFS | 5,10-Methenyltetrahydrofolate synthetase |
| MTR | Methionine synthase |
| MTRR | Methionine synthase reductase |
| MUT | O-6-Methylguanine-DNA Methyltransferase |
| NAT1 | N-Acetyltransferase 1 |
| NAT2 | N-Acetyltransferase 2 |
| NNMT | Nicotinamide N-Methyltransferase |
| PEMT | Nicotinamide N-Methyltransferase |
| PRMT1 | Protein Arginine Methyltransferase 1 |
| PRMT2 | Protein Arginine Methyltransferase 2 |
| PRMT5 | Protein Arginine Methyltransferase 5 |
| RNMT | RNA Guanine-7 Methyltransferase |
| SARDH | Sarcosine Dehydrogenase |
| SHMT1 | Serine Hydroxymethyltransferase 1 |
| SLC19A1 | Reduced folate carrier |
| TCN2 | Transcobalamin II |
| TRDMT1 | TRNA Aspartic Acid Methyltransferase 1 |
| TYMS | Thymidylate Synthetase |
